# Daily Stress, Cortisol Dysregulation, and Screen Time in Midlife and Older Adults: Findings from the MIDUS Study

**DOI:** 10.1093/geroni/igaf122.3065

**Published:** 2025-12-31

**Authors:** Rinanda Shaleha, David Almeida, Nelson Roque

**Affiliations:** The Pennsylvania State University, University Park, Pennsylvania, United States; The Pennsylvania State University, University Park, Pennsylvania, United States; The Pennsylvania State University, University Park, Pennsylvania, United States

## Abstract

As digital engagement increases, its relationship with daily psychological stress and physiological regulation in midlife and older adults remains underexplored. Guided by the Strength and Vulnerability Integration (SAVI) model, this study investigated whether daily stress and cortisol dysregulation predict screen time behaviors, specifically social media use and TV watching using data from the Midlife in the United States (MIDUS) Refresher 1. 542 participants (Mage = 54.53, SD = 9.53) completed 8-day daily diaries, and a subset provided cortisol data across 4 days. Separate multilevel models tested within-person associations, adjusting for age, sex, self-rated health, chronic conditions, and weekday/weekend variation. Same-day stress was not associated with social media use (β = -0.59, p = .705) or TV watching (β = -4.75, p = .114). However, higher stress predicted marginally less TV viewing the next day (β = -5.64, p < .05). A flatter cortisol slope also predicted reduced next-day TV time (β = -0.08, p < .05), though the effect was small, consistent with subtle physiological influences on behavior. Same-day cortisol effects were not significant. Poor self-rated health was consistently linked to lower screen time. Older adults watched more TV overall (same-day: β = 2.17; next-day: β = 1.63, p < .01), but age did not moderate stress or cortisol effects. Findings suggest that next-day disengagement from TV follows heightened psychological or physiological stress, aligning with SAVI’s view that older adults regulate emotions but remain stress-sensitive. This may inform strategies for balanced media use in aging populations.

